# Supplementary material for: Modeling glioblastoma heterogeneity as a dynamic network of cell states
Source: Mol Syst Biol. 2021 Sep 16;17(9):e10105. doi: 10.15252/msb.202010105 (PMC8444284; doi:10.15252/msb.202010105)
Supplement: Supplementary file 6 — Source Data for Figure 5 [file MSB-17-e10105-s004.zip › Figure5A_sourcedata/GSEA_3071/hallmarks_stateVI.GseaPreranked.1623742557031/HALLMARK_APOPTOSIS.html]

Details for gene set HALLMARK\_APOPTOSIS[GSEA]

|  || Dataset | state63071 |
| Phenotype | NoPhenotypeAvailable |
| Upregulated in class | na\_neg |
| GeneSet | HALLMARK\_APOPTOSIS |
| Enrichment Score (ES) | -0.27382642 |
| Normalized Enrichment Score (NES) | -1.2894815 |
| Nominal p-value | 0.18223235 |
| FDR q-value | 0.40471622 |
| FWER p-Value | 0.998 |
Table: GSEA Results Summary

  

Fig 1: Enrichment plot: HALLMARK\_APOPTOSIS      
 Profile of the Running ES Score & Positions of GeneSet Members on the Rank Ordered List

  

| PROBE | GENE SYMBOL | GENE\_TITLE | RANK IN GENE LIST | RANK METRIC SCORE | RUNNING ES | CORE ENRICHMENT || 1 | CAV1 |  |  | 13 | 1.087 | 0.0242 | No |
| 2 | DDIT3 |  |  | 14 | 1.086 | 0.0520 | No |
| 3 | GADD45B |  |  | 90 | 0.758 | 0.0504 | No |
| 4 | SQSTM1 |  |  | 147 | 0.667 | 0.0518 | No |
| 5 | BIRC3 |  |  | 191 | 0.608 | 0.0553 | No |
| 6 | GADD45A |  |  | 200 | 0.603 | 0.0685 | No |
| 7 | IL18 |  |  | 239 | 0.572 | 0.0725 | No |
| 8 | BCL2L1 |  |  | 247 | 0.567 | 0.0851 | No |
| 9 | PMAIP1 |  |  | 255 | 0.561 | 0.0975 | No |
| 10 | JUN |  |  | 321 | 0.527 | 0.0927 | No |
| 11 | HSPB1 |  |  | 322 | 0.527 | 0.1062 | No |
| 12 | IGFBP6 |  |  | 341 | 0.515 | 0.1144 | No |
| 13 | LMNA |  |  | 383 | 0.496 | 0.1156 | No |
| 14 | TXNIP |  |  | 402 | 0.487 | 0.1230 | No |
| 15 | ATF3 |  |  | 416 | 0.483 | 0.1318 | No |
| 16 | GPX3 |  |  | 493 | 0.457 | 0.1222 | No |
| 17 | DFFA |  |  | 623 | 0.419 | 0.0967 | No |
| 18 | TNFSF10 |  |  | 631 | 0.416 | 0.1054 | No |
| 19 | ETF1 |  |  | 715 | 0.393 | 0.0923 | No |
| 20 | IRF1 |  |  | 771 | 0.382 | 0.0866 | No |
| 21 | PAK1 |  |  | 833 | 0.371 | 0.0790 | No |
| 22 | DAP3 |  |  | 901 | 0.361 | 0.0695 | No |
| 23 | BMP2 |  |  | 940 | 0.354 | 0.0679 | No |
| 24 | VDAC2 |  |  | 1025 | 0.341 | 0.0531 | No |
| 25 | CCND2 |  |  | 1064 | 0.335 | 0.0510 | No |
| 26 | TIMP3 |  |  | 1290 | 0.305 | -0.0042 | No |
| 27 | PSEN2 |  |  | 1442 | 0.286 | -0.0392 | No |
| 28 | KRT18 |  |  | 1570 | 0.272 | -0.0678 | No |
| 29 | PLCB2 |  |  | 1655 | 0.264 | -0.0846 | No |
| 30 | ISG20 |  |  | 1756 | 0.255 | -0.1061 | No |
| 31 | RELA |  |  | 1791 | 0.252 | -0.1092 | No |
| 32 | TGFBR3 |  |  | 1846 | -0.256 | -0.1178 | No |
| 33 | SAT1 |  |  | 1952 | -0.271 | -0.1402 | No |
| 34 | CASP6 |  |  | 1996 | -0.278 | -0.1452 | No |
| 35 | PTK2 |  |  | 2347 | -0.343 | -0.2345 | No |
| 36 | BAX |  |  | 2397 | -0.353 | -0.2392 | No |
| 37 | CASP2 |  |  | 2404 | -0.353 | -0.2318 | No |
| 38 | F2R |  |  | 2478 | -0.372 | -0.2427 | No |
| 39 | ADD1 |  |  | 2529 | -0.384 | -0.2469 | No |
| 40 | MMP2 |  |  | 2550 | -0.388 | -0.2426 | No |
| 41 | BGN |  |  | 2551 | -0.388 | -0.2326 | No |
| 42 | CASP3 |  |  | 2699 | -0.426 | -0.2629 | Yes |
| 43 | CREBBP |  |  | 2726 | -0.432 | -0.2592 | Yes |
| 44 | CDK2 |  |  | 2746 | -0.439 | -0.2532 | Yes |
| 45 | LUM |  |  | 2775 | -0.448 | -0.2496 | Yes |
| 46 | IGF2R |  |  | 2838 | -0.469 | -0.2550 | Yes |
| 47 | IFITM3 |  |  | 2851 | -0.477 | -0.2461 | Yes |
| 48 | CTNNB1 |  |  | 2863 | -0.483 | -0.2369 | Yes |
| 49 | SATB1 |  |  | 2899 | -0.494 | -0.2340 | Yes |
| 50 | CFLAR |  |  | 2902 | -0.495 | -0.2219 | Yes |
| 51 | GPX4 |  |  | 2913 | -0.499 | -0.2119 | Yes |
| 52 | DPYD |  |  | 2940 | -0.510 | -0.2062 | Yes |
| 53 | WEE1 |  |  | 2974 | -0.527 | -0.2019 | Yes |
| 54 | DCN |  |  | 3027 | -0.554 | -0.2023 | Yes |
| 55 | GSN |  |  | 3041 | -0.559 | -0.1917 | Yes |
| 56 | ERBB3 |  |  | 3116 | -0.596 | -0.1971 | Yes |
| 57 | LEF1 |  |  | 3149 | -0.616 | -0.1903 | Yes |
| 58 | TIMP2 |  |  | 3180 | -0.643 | -0.1822 | Yes |
| 59 | BTG3 |  |  | 3184 | -0.647 | -0.1665 | Yes |
| 60 | BRCA1 |  |  | 3335 | -0.802 | -0.1880 | Yes |
| 61 | TGFB2 |  |  | 3340 | -0.804 | -0.1685 | Yes |
| 62 | BTG2 |  |  | 3377 | -0.857 | -0.1567 | Yes |
| 63 | CTH |  |  | 3401 | -0.894 | -0.1402 | Yes |
| 64 | HMGB2 |  |  | 3414 | -0.922 | -0.1200 | Yes |
| 65 | PLAT |  |  | 3416 | -0.927 | -0.0965 | Yes |
| 66 | FEZ1 |  |  | 3423 | -0.945 | -0.0740 | Yes |
| 67 | CDKN1B |  |  | 3426 | -0.951 | -0.0502 | Yes |
| 68 | TOP2A |  |  | 3457 | -1.020 | -0.0325 | Yes |
| 69 | APP |  |  | 3562 | -1.427 | -0.0251 | Yes |
| 70 | CLU |  |  | 3601 | -1.801 | 0.0104 | Yes |
Table: GSEA details [plain text format]

  

Fig 2: HALLMARK\_APOPTOSIS: Random ES distribution      
 Gene set null distribution of ES for **HALLMARK\_APOPTOSIS**
